# Paediatric research in the times of COVID-19

**DOI:** 10.1038/s41390-021-01479-6

**Published:** 2021-04-20

**Authors:** Paul F. Fleming, Chris Gale, Eleanor J. Molloy, Saul N. Faust, Kate Costeloe, Edmund Juszczak, Charles C. Roehr

**Affiliations:** 1 Homerton University Hospital, NHS Foundation Trust, London, UK; 2 Centre for Genomics and Child Health, Queen Mary University of London, London, UK; 3 Neonatal Medicine, School of Public Health, Faculty of Medicine, Imperial College London, London, UK; 4 Chelsea and Westminster Hospital NHS Foundation Trust, London, UK; 5 Discipline of Paediatrics, Trinity Translational Medicine Institute (TTMI) & Trinity Research in Childhood Centre (TRiCC), Trinity College, The University of Dublin, Dublin, Ireland; 6 Neonatology, Coombe Women and Infant’s University Hospital, Dublin, Ireland; 7 Neonatology, Children’s Hospital Ireland (CHI) at Crumlin & Tallaght, Dublin, Ireland; 8 NIHR Southampton Clinical Research Facility and Biomedical Research Centre, Southampton University Hospital NHS Foundation Trust; and Faculty of Medicine and Institute for Life Sciences, University of Southampton, Southampton, UK; 9 Nottingham Clinical Trials Unit, University of Nottingham, Nottingham, UK; 10 Nuffield Department of Population Health, National Perinatal Epidemiology Unit, Medical Sciences Division, University of Oxford, Oxford, UK; 11 Newborn Services, John Radcliffe Hospital, Oxford University Hospitals, Oxford, UK

## Abstract

**Abstract:**

The COVID-19 pandemic poses many direct and indirect consequences for children’s health and associated research. Direct consequences include participation of children in COVID-19 research trials, pausing other research in children and the potential implications of a global economic downturn on future research funding. Collaborative and networked research together with streamlined research processes and use of remote technology have been central to efforts by clinicians and scientists around the world and have proved essential for reducing COVID-19 morbidity and mortality.

**Impact:**

- Maintain streamlined and efficient approaches to research governance and data sharing to facilitate high-quality collaborative research.
- Ensure early inclusion of children in trials of therapies for diseases that affect all age groups.
- Paediatric Research Societies should co-ordinate effective processes to define key research questions and develop multinational clinical trials for diagnostics, therapeutics and preventative strategies for infants, children and young people.

## Introduction

As coronavirus disease 2019 (COVID-19) spread across the globe in early 2020, healthcare systems adapted urgently to respond to and understand this newly emerging, highly infectious disease. COVID-19 in its symptomatic and most severe form primarily affects adults, particularly the elderly and those with underlying health conditions.^1,2^ Children are less severely affected representing <5% of cases.^3–7^ However, in May 2020, a severe post-infectious complication of COVID-19 in children, the paediatric inflammatory multisystem syndrome temporally associated with COVID-19 (PIMS-TS) [also known as multisystem inflammatory syndrome in children and adolescents temporally associated with COVID-19] was described^8^ and is now recognised as a significant cause of COVID-19-associated morbidity.^9^

Although the incidence of symptomatic and of severe COVID-19 is lower in children, the current pandemic has an increasing number of implications for children’s healthcare and associated research. This opinion piece explores some of the effects that the global pandemic has had upon both COVID-19 and non-COVID-19 research in children, including inclusion of children in COVID-19 research studies and the importance of research delivery through established research networks, how streamlined research approvals have facilitated rapid evaluations of potential treatments, impacts of pausing non-COVID-19 research in children and availability and reconfiguration of post-pandemic research funding and what this might mean for children’s research in the future. We also looked at some of the positive research developments and innovations consequent on COVID-19 and recommend improvements and modifications we should strive to make as a lasting beneficial legacy of the pandemic.

## Responding to a global pandemic

Despite previous warnings, when COVID-19 first emerged, pandemic disaster preparedness across the world was insufficient^10^ as was delivery of children’s health priorities.^11^ Global scientific and medical efforts mobilised quickly. These focussed on epidemiology^2^ and therapeutic trials to understand the burden of disease and to find effective treatments for those individuals who were critically unwell.^12^ Simultaneously, a range of studies was undertaken aimed at better understanding the immune response to infection,^13^ developing and evaluating diagnostic tests and treatments that might dampen the harmful inflammatory responses,^14^ developing vaccines directed towards finding a longer-term solution,^15^ evaluating and comparing guidance on the appropriate use of personal protective equipment,^16,17^ identifying novel COVID-19 variants and assessing their transmissibility^18^ and evaluating vaccine efficacy against variants.^19^ During the first wave, swathes of research funding and personnel were redeployed to COVID-19 research studies and, in many cases, the provision of frontline care. Within children’s healthcare, a number of international collaborations were quickly formed to share information and optimise safe delivery of care to children.^20–22^

## Children’s inclusion in COVID-19 research, vaccine evaluations and the role of research networks

The general assumption that children are less severely affected by COVID-19 combined with residual historic reluctance to include children and pregnant women in the early stages of therapeutic trials has contributed to delays in including these groups in COVID-19 trials.^23,24^ This has interrupted evidence-based evaluations of potential COVID-19 treatments for hospitalised children and for those who present with more severe forms of the disease. Instead, a standardised approach should be encouraged that best serves the needs of all patients while systematically improving our understanding of the effectiveness and safety of interventions.^25,26^

Recognising the need for children to have access to the same or similar treatments as adults, children in the United Kingdom were included in the RECOVERY (Randomised Evaluation of COVID-19 Therapy) trial. The RECOVERY trial is an adaptive multi-arm multicentre platform trial evaluating potential treatments for COVID-19^27^ and was set up in 9 days, began recruiting patients in March 2020 and was rolled out in 174 hospitals throughout the UK. By day 16 of recruitment, the 1000th patient was recruited and by March 2021, >38,000. With some age-specific protocol modifications (Version 5), children with respiratory COVID-19 infection were included from May 2020 and have been allocated to corticosteroid, antiviral, convalescent plasma and immunomodulatory arms of this study. Following the emergence of PIMS-TS,^28^ the RECOVERY trial protocol was adapted in July 2020 to evaluate potential treatments for this condition^29^ using a standardised approach.^30^ At present, the PIMS-TS arm of the RECOVERY trial is evaluating corticosteroid and intravenous immunoglobulin for initial therapies and tocilizumab and anakinra for children requiring additional immunomodulatory therapy.

By June 2020, analyses of three principal comparisons in RECOVERY provided crucial findings on repurposed treatments for COVID-19, including dexamethasone,^12^ hydroxychloroquine^31^ and lopinavir–ritonavir.^32^ Subsequent findings on azithromycin^33^ and convalescent plasma have also been reported.^27^ Studies like the RECOVERY trial are necessary to safely evaluate COVID-19 interventions in children but are few and far between. As of January 2021, <10% of registered interventional studies in COVID-19 on clinicaltrials.gov included children.

Although children were initially involved in the first protocols to test pandemic vaccines, the emergence of PIMS-TS made paediatric vaccine researchers and pharmaceutical companies delay paediatric enrolment due to concerns that PIMS-TS pathophysiology may have an antibody-driven component. There are now large-scale safety data for the first three vaccines to protect against COVID-19 under evaluation by global regulatory authorities, none of which shows any suggestion of inflammatory adverse effects in adults.^34–36^ Several COVID-19 vaccines are due to start specific paediatric and teenage dose-finding and safety trials, together with trials in pregnant women, in early 2021. It is important that research systems and networks give equal priority to these trials. Regulatory authorities will need to consider whether resource-consuming large-scale phase 3 efficacy trials are necessary once individual vaccines have identified paediatric dosing and initial safety data in smaller cohorts. To try to minimise global education disruption in the immediate years to come, Phase 4 safety trials during large-scale deployment may be more appropriate, especially if adult trials identify as-yet, unknown immunological correlates of protection.

Clinical research networks have played an important role throughout the pandemic. The World Health Organisation (WHO) ISARIC standardised data collection protocol,^37^ the neonatal COVID-19 networks^20^ and British Paediatric Surveillance Unit^6^ are some examples of how networks have provided crucial epidemiological data identifying at-risk patient groups, informing best clinical practice and identifying priority areas for critical research delivery. Deployment of studies like the RECOVERY trial is testament to the UK’s clinical research network infrastructure^38^ and well-established public healthcare system, which allow patient groups across all ages access to clinical research priority studies.

## Streamlined research approval

Alongside light-touch streamlined systems that allow for rapid patient recruitment, consent, randomisation and data collection,^39^ research systems have evolved even more efficient risk-based systems for research approval and delivery. The speedy delivery of studies like the RECOVERY trial was enhanced by embedding the research in clinical practice, with simple, targeted but effective staff training and site governance requirements.

Research governance and patient safety are critical but approvals often have multiple layers of bureaucracy that inevitably slow the rate at which studies are conducted. In response to COVID-19 treatment evaluations, many countries have introduced streamlined approaches to approve research while using remote video conferencing to convene relevant meetings, such as ethics review boards. As we move forward, we should learn how the approval processes can be better aligned to deliver efficient and proportionate regulation while ensuring current levels of patient safety are maintained. This may require a fundamental re-evaluation and risk analysis of the way research is conducted alongside routine clinical care. Research training and governance can certainly be better targeted in a risk-appropriate way, with more resource and oversight for complex or higher-risk early phase trials and later phase trials more embedded in routine clinical care.^40^

## Impact of pausing children’s research

In response to the urgency and severity of the COVID-19 pandemic, many countries and healthcare systems initially paused non-COVID-19 clinical research studies.^26^ Despite children being less severely affected by COVID-19, this pause involved important studies cross all paediatric domains including many that had taken years to establish. There is also an indirect financial burden from pausing research if this results in delayed dissemination of results that have potential cost-saving implications.^41^ In retrospect, these pauses may have disproportionately and negatively affected children and may have arisen from gaps in preparedness and limited learning from previous pandemics^11^ together with uncertainties about the evolution of the COVID-19 pandemic.

Furthermore, COVID-19 fundamentally changed the context of many patient-facing clinical and translational research efforts as a result of plummeting rates of admission for commonly studied conditions.^42^ A reduced disease burden together with limited research opportunities could derail the research aspirations of young investigators at a critical point in their career development and deprive them of the opportunities to conduct research. This, in turn, may impact the critical mass of experienced paediatric researchers in the future.

Alternative strategies for maintaining research have been proposed, including modifications to the consent process, prioritisation of outcome data collection and exploring alternative methods of measuring key outcomes (e.g. continuing data collection remotely or online).^43^ Such strategies need monitoring as their impact on the validity of studies remains unclear. Before 2020, contingency measures to account for such a global pandemic were highly unlikely to be the focus for many researchers. This is in itself an important lesson in terms of risk assessment.

When the time came to restart children’s research, the initial focus was on studies that were already recruiting prior to the pandemic, quick and efficient progression of those studies already ‘in set-up’ and supporting paediatric research trainees whose projects were suspended by the pandemic and who have limited time in which to complete their studies. Where possible, this was done using mechanisms that reduced administration time and costs, thereby limiting the negative impact of the pause on research.

As we re-start research, consideration of the wider impact of COVID-19 on children’s health needs to be evaluated and researched. There are many areas within children’s health that are under ongoing scrutiny such as the impacts of isolation on mental health, child protection issues, COVID-19 severity in children with chronic diseases, reduced attendance at hospitals,^44^ healthcare disparities and how some of these disparities might be mitigated through better access to telehealth.^45^ These important areas of research need further support so that we can understand and address the impact of COVID-19 on health and wellbeing and are ready to respond better to future pandemics.

## Post-pandemic research funding reconfiguration and implications for future children’s research

Expectedly and appropriately, the pandemic has resulted in a rapid realignment and unparalleled focus on COVID-19 research. Effective treatment and prevention of COVID-19 are essential at an individual and societal level, as a healthy workforce is the cornerstone of a thriving economy, which in turn underpins current and future research activity. Most research funders have acted rapidly and flexibly to support existing research impacted by COVID-19. However, the funding landscape for future research, particularly research not focused on COVID-19, is highly uncertain, at least in the short to medium term. Charity funding for health research has been severely affected by the pandemic. One example is The Association of Medical Research Charities in the UK, who warned that their ability to fund research may fall by over £300 million in 2020 as a result of the impact of COVID-19 on fundraising activities.^46^ This will have widespread impacts on fundamental research, clinical trials, research infrastructure and on research fellowships and other individual support at all career stages. Furthermore, the pandemic has caused a significant contraction in the global economy, which will almost certainly lead to lower government funding of medical research in some countries.

Such systematic changes to the research funding landscape will inevitably impact paediatric research, which was already under-represented relative to other age groups and disease areas prior to COVID-19.^47,48^ Children have been disproportionately affected by responses to the COVID-19 pandemic, for instance by school closures in lockdown and entire year groups or “bubbles” spending time in self-isolation due to case identification in schools when open. To ensure that paediatric research is not overly impacted post-pandemic, we, as paediatric researchers and clinician scientists, must advocate for the importance of such research to improve the future health and wealth of nations. The UK Children’s Charter highlights the importance of equity between children’s and adult research and now, more than ever, preventing conditions such as obesity and hypertension in childhood is particularly pertinent as these conditions predispose to severe disease among adults affected by COVID-19 (https://www.rcpch.ac.uk/resources/research-charter-infants-childrens-young-peoples-child-health).

## What are the positive outcomes to emerge from the COVID-19 pandemic?

Several positive outcomes for children’s research have arisen during this period. One has been recognition from across the research spectrum that rapid, responsive collaboration can be achieved and is essential to effectively and quickly address important clinical questions. Examples of these collaborations include epidemiological studies allowing better understanding of COVID-19 infection in children,^5^ defining core outcomes for assessment in clinical trials so that meaningful streamlined data can be collected^49,50^ and for sharing and collating guidelines for specific populations, such as newborn babies.^20,51^ These global collaborations are a credit to the efforts of researchers to bring knowledge and best available practice together and to disseminate it quickly and efficiently via social media and online collaboration.^52^

The second is the recognition that study design may not need multiple complex layers of administration and that approval processes can be streamlined without compromising quality and safety. Necessary remote working and use of video conferencing technology has allowed people to become familiar with conducting meetings, delivering teaching, attending seminars and even conferences from multiple locations across the world. The online summer series provided by the Pediatric Academic Society or the fully virtual European Academic Paediatric Societies meeting are just two examples of how important forums for researchers can be adapted to facilitate dissemination and discussion of results from clinical trials and research studies remotely to influence current clinical practice within a global pandemic.

The use of technology has facilitated more rapid identification platforms and early warning systems for emerging diseases like PIMS-TS and allowed paediatric experts from across the world to collaboratively and quickly reach agreed consensus on case definitions and treatment protocols.^50^ Routinely collected electronic patient data with standardised definitions of outcomes are used increasingly to compare neonatal services^53^ and access to such data that are not dependent on conventional, often cumbersome, methods of data collection paves the way to streamlining data collection for clinical trials. Finally, the success of research networks may allow us to reconsider how best we can collaborate and establish international paediatric networks to address clinically important questions about children’s health and disease and thereby accelerate recruitment and generalisability of results.

One of the most important outcomes from this pandemic relates to the widespread rollout of novel vaccines, which will hopefully prove safe and effective in ongoing and future paediatric trials.^54^ Furthermore, during COVID-19 vaccine research, local networks and pharmaceutical companies have established new systems such as allowing one organisation (one legal entity) to handle all contracting and governance for contract commercial research protocols opening in multiple sites and organisations. Such systems have proved efficient and cost-effective and should be a lasting benefit of the pandemic on the partnership between industry and health systems. COVID-19 experience has shown that contract commercial research organisations will need to adapt or be rapidly left behind in the emerging post-COVID-19 world.

The European Society for Paediatric Research (ESPR) and the Society for Pediatric Research are examples of hubs where paediatric clinical researchers meet, share and disseminate research findings. The ESPR has advanced numerous paediatric COVID-19 research collaborations and research mentoring schemes (www.espr.eu). Thus, societies have taken a pivotal role in forging and fostering international collaborations to coordinate bench-top research and acute COVID-19 clinical guideline development and knowledge exchange from the start of the pandemic.^20,52,55–57^ In light of the current pandemic and in preparation for the next, such societies are well placed to become perhaps the most important providers of platforms for forming such networks and for coordinating public patient involvement.^58,59^

## Will the COVID-19 pandemic affect the way we conduct children’s research in the future?

Social science often uses the terms ‘generation’ and ‘birth cohorts’ synonymously to refer to ‘*people within a delineated population who experience the same significant events within a given time period*’.^60^ Historically these ‘generations’ include the Baby Boomers, Generation X and Millennials and perhaps now, in the context of the current global pandemic, Generation COVID (Gen C). Generation C is likely to experience a wide variety of health consequences. These consequences will inevitably influence the way we conduct children’s research in the future. Platform trials might be a means by which pragmatic trials are conducted in certain paediatric populations, such as newborn babies. We should ensure that we shape health research agendas now so that children remain at the forefront of current clinical research priorities. In order to achieve the right outcomes for children, paediatric researchers should harness the positive advances while leaving behind the negative impact this pandemic has exerted on children’s research.

## Conclusion

With encouraging updates from potentially successful vaccines against COVID-19,^61–63^ a path to the end of this pandemic is visible. To achieve better outcomes for children’s healthcare research, even stronger international networks and collaborations need to be developed and fostered. Paediatric researchers must continue to raise the profile of their research, to streamline research design and approvals and to create new relationships and ways of working with industry to allow fast and pragmatic deployment of research to address the ever-important health needs of children. In doing so, the positive outcomes learned during this pandemic can create a better platform for children’s research as we move forward.

## References

[1] de Lusignan, S. et al. Risk factors for SARS-CoV-2 among patients in the Oxford Royal College of General Practitioners Research and Surveillance Centre primary care network: a cross-sectional study. *Lancet Infect. Dis*. **20**, 1034–1042 (2020). 10.1016/S1473-3099(20)30371-6 PMC7228715 32422204

[2] Docherty AB (2020). Features of 20 133 UK patients in hospital with covid-19 using the ISARIC WHO Clinical Characterisation Protocol: prospective observational cohort study. BMJ.

[3] Wu, Z. & McGoogan, J. M. Characteristics of and important lessons from the coronavirus disease 2019 (COVID-19) outbreak in China: summary of a report of 72314 cases from the Chinese Center for Disease Control and Prevention. *JAMA* **323**, 1239–1242 (2020). 10.1001/jama.2020.2648 32091533

[4] Tagarro, A. et al. Screening and severity of coronavirus disease 2019 (COVID-19) in children in Madrid, Spain. *JAMA Pediatr*. 10.1001/jamapediatrics.2020.1346 (2020). 10.1001/jamapediatrics.2020.1346 PMC7142799 32267485

[5] Gotzinger F (2020). COVID-19 in children and adolescents in Europe: a multinational, multicentre cohort study. Lancet Child Adolesc. Health.

[6] Gale, C. et al. Characteristics and outcomes of neonatal SARS-CoV-2 infection in the UK: a prospective national cohort study using active surveillance. *Lancet Child Adolesc. Health* **5**, 113–121 (2021). 10.1016/S2352-4642(20)30342-4 PMC7818530 33181124

[7] Swann OV (2020). Clinical characteristics of children and young people admitted to hospital with covid-19 in United Kingdom: prospective multicentre observational cohort study. BMJ.

[8] Davies P (2020). Intensive care admissions of children with paediatric inflammatory multisystem syndrome temporally associated with SARS-CoV-2 (PIMS-TS) in the UK: a multicentre observational study. Lancet Child Adolesc. Health.

[9] Carter, M. J. et al. Peripheral immunophenotypes in children with multisystem inflammatory syndrome associated with SARS-CoV-2 infection. *Nat. Med*. **26**, 1701–1707 (2020). 10.1038/s41591-020-1054-6 32812012

[10] Gates B (2015). The next epidemic-lessons from Ebola. N. Engl. J. Med..

[11] Nicholas DB (2020). Perceived impacts of the COVID-19 pandemic on pediatric care in Canada: a roundtable discussion. Glob. Pediatr. Health.

[12] RECOVERY Collaborative Group et al. Dexamethasone in hospitalized patients with Covid-19 - preliminary report. *N. Engl. J. Med*. **384**, 693–704 (2021). 10.1056/NEJMoa2021436 PMC7383595 32678530

[13] Laing, A. G. et al. A dynamic COVID-19 immune signature includes associations with poor prognosis. *Nat. Med*. **26**, 1663 (2020). 10.1038/s41591-020-1079-x PMC7479399 32908251

[14] Luo P (2020). Tocilizumab treatment in COVID-19: a single center experience. J. Med. Virol..

[15] Folegatti, P. M. et al. Safety and immunogenicity of the ChAdOx1 nCoV-19 vaccine against SARS-CoV-2: a preliminary report of a phase 1/2, single-blind, randomised controlled trial. *Lancet* **396**, 467–478 (2020). 10.1016/S0140-6736(20)31604-4 PMC7445431 32702298

[16] Ferioli, M. et al. Protecting healthcare workers from SARS-CoV-2 infection: practical indications. *Eur. Respir. Rev*. **29**, 200068 (2020). 10.1183/16000617.0068-2020 PMC7134482 32248146

[17] Verbeek JH (2020). Personal protective equipment for preventing highly infectious diseases due to exposure to contaminated body fluids in healthcare staff. Cochrane Database Syst. Rev..

[18] Volz E (2021). Evaluating the effects of SARS-CoV-2 spike mutation D614G on transmissibility and pathogenicity. Cell.

[19] Xie, X. et al. Neutralization of SARS-CoV-2 spike 69/70 deletion, E484K and N501Y variants by BNT162b2 vaccine-elicited sera. *Nat. Med*. 10.1038/s41591-021-01270-4 (2021). 10.1038/s41591-021-01270-4 33558724

[20] Lavizzari, A. et al. International comparison of guidelines for managing neonates at the early phase of the SARS-CoV-2 pandemic. *Pediatr. Res*. 10.1038/s41390-020-0976-5 (2020). 10.1038/s41390-020-0976-5 32541844

[21] Klein JD (2020). Promoting and supporting children’s health and healthcare during COVID-19 - International Paediatric Association Position Statement. Arch. Dis. Child..

[22] Kache S (2020). COVID-19 PICU guidelines: for high- and limited-resource settings. Pediatr. Res..

[23] Malhotra, A., Kumar, A., Roehr, C. C. & den Boer, M. C. Inclusion of children and pregnant women in COVID-19 intervention trials. *Pediatr. Res*. 10.1038/s41390-020-1067-3 (2020). 10.1038/s41390-020-1067-3 32688370

[24] Hwang TJ, Randolph AG, Bourgeois FT (2020). Inclusion of children in clinical trials of treatments for coronavirus disease 2019 (COVID-19). JAMA Pediatr..

[25] Zagury-Orly I, Schwartzstein RM (2020). Covid-19 - a reminder to reason. N. Engl. J. Med..

[26] McDermott, M. M. & Newman, A. B. Preserving clinical trial integrity during the coronavirus pandemic. *JAMA* **323**, 2135–2136 (2020). 10.1001/jama.2020.4689 32211830

[27] Nuffield Department of Population Health. Welcome—RECOVERY Trial. https://www.recoverytrial.net/ (2020).

[28] Davies, P. et al. Intensive care admissions of children with paediatric inflammatory multisystem syndrome temporally associated with SARS-CoV-2 (PIMS-TS) in the UK: a multicentre observational study. *Lancet Child Adolesc. Health* **4**, 669–677 (2020). 10.1016/S2352-4642(20)30215-7 PMC7347350 32653054

[29] Nuffield Department of Population Health. Information for site staff. https://www.recoverytrial.net/for-site-staff (2020).

[30] Bourgeois, F. T., Avillach, P. & Turner, M. A. The urgent need for research coordination to advance knowledge on COVID-19 in children. *Pediatr. Res*. 10.1038/s41390-020-01259-8 (2020). 10.1038/s41390-020-01259-8 PMC7656217 33177674

[31] The RECOVERY Collaborative Group. Effect of hydroxychloroquine in hospitalized patients with Covid-19. *N. Engl. J. Med*. **383**, 2030–2040 (2020). 10.1056/NEJMoa2022926 PMC7556338 33031652

[32] RECOVERY Collaborative Group. Lopinavir-ritonavir in patients admitted to hospital with COVID-19 (RECOVERY): a randomised, controlled, open-label, platform trial. *Lancet* **396**, 1345–1352 (2020). 10.1016/S0140-6736(20)32013-4 PMC7535623 33031764

[33] RECOVERY Collaborative Group. (2021). Azithromycin in patients admitted to hospital with COVID-19 (RECOVERY): a randomised, controlled, open-label, platform trial. Lancet.

[34] Pfizer. Pfizer and BioNTech announce vaccine candidate against COVID-19 achieved success in first interim analysis from phase 3 study. https://www.pfizer.com/news/press-release/press-release-detail/pfizer-and-biontech-announce-vaccine-candidate-against (2020).

[35] Moderna. Moderna announces primary efficacy analysis in phase 3 COVE study for its COVID-19 vaccine candidate and filing today with U.S. FDA for emergency use authorization. https://investors.modernatx.com/news-releases/news-release-details/moderna-announces-primary-efficacy-analysis-phase-3-cove-study (2020).

[36] AstraZeneca. AZD1222 vaccine met primary efficacy endpoint in preventing COVID-19. https://www.astrazeneca.com/media-centre/press-releases/2020/azd1222hlr.html (2020).

[37] ISARIC. Covid-19 clinical research resources. https://isaric.org/research/covid-19-clinical-research-resources/ (2020).

[38] Lythgoe H (2017). NIHR Clinical Research Networks: what they do and how they help paediatric research. Arch. Dis. Child..

[39] Wilkinson E (2020). RECOVERY trial: the UK covid-19 study resetting expectations for clinical trials. BMJ.

[40] Mitchell EJ (2020). It is unprecedented: trial management during the COVID-19 pandemic and beyond. Trials.

[41] Marshall ASJ (2020). Study protocol: NeoCLEAR: Neonatal Champagne Lumbar punctures Every time - An RCT: a multicentre, randomised controlled 2 x 2 factorial trial to investigate techniques to increase lumbar puncture success. BMC Pediatr..

[42] Yeoh, D. K. et al. The impact of COVID-19 public health measures on detections of influenza and respiratory syncytial virus in children during the 2020 Australian winter. *Clin. Infect. Dis*. 10.1093/cid/ciaa1475 (2020). 10.1093/cid/ciaa1475 PMC7543326 32986804

[43] Balevic, S. J. et al. Bringing research directly to families in the era of COVID-19. *Pediatr. Res*. 10.1038/s41390-020-01260-1 (2020). 10.1038/s41390-020-01260-1 PMC7656893 33177676

[44] Lachman, P. Where to make a difference: research and the social determinants in pediatrics and child health in the COVID-19 era. *Pediatr. Res*. **89**, 259–262 (2021). 10.1038/s41390-020-01253-0 PMC7654351 33173180

[45] Menon, D. U. & Belcher, H. M. E. COVID-19 pandemic health disparities and pediatric health care-the promise of telehealth. *JAMA Pediatr*. 10.1001/jamapediatrics.2020.5097 (2020). 10.1001/jamapediatrics.2020.5097 33284346

[46] AMRC. COVID-19: the risk to AMRC charities. https://www.amrc.org.uk/covid-19-the-risk-to-amrc-charities (2020).

[47] Gitterman DP, Langford WS, Hay WW (2018). The fragile state of the National Institutes of Health Pediatric Research Portfolio, 1992-2015: doing more with less?. JAMA Pediatr..

[48] Molloy EJ (2017). The future of pediatric research: European perspective. Pediatr. Res..

[49] Tong, A. et al. Core outcomes set for trials in people with coronavirus disease 2019. *Crit. Care Med*. **48**, 1622–1635 (2020). 10.1097/CCM.0000000000004585 PMC7448717 32804792

[50] Harwood, R. et al. A national consensus management pathway for paediatric inflammatory multisystem syndrome temporally associated with COVID-19 (PIMS-TS): results of a national Delphi process. *Lancet Child Adolesc. Health* **5**, 133–141 (2020). 10.1016/S2352-4642(20)30304-7 PMC7500943 32956615

[51] Yeo, K. T. et al. Review of guidelines and recommendations from 17 countries highlights the challenges that clinicians face caring for neonates born to mothers with COVID-19. *Acta Paediatr*. **109**, 2192–2207 (2020). 10.1111/apa.15495 32716579

[52] Molloy, E. J. et al. Neonates in the COVID-19 pandemic. *Pediatr. Res*. 10.1038/s41390-020-1096-y (2020). 10.1038/s41390-020-1096-y 32746446

[53] Lui K (2019). Trends in outcomes for neonates born very preterm and very low birth weight in 11 high-income countries. J. Pediatr..

[54] Pardi N, Hogan MJ, Porter FW, Weissman D (2018). mRNA vaccines - a new era in vaccinology. Nat. Rev. Drug Discov..

[55] Terheggen, U. et al. European consensus recommendations for neonatal and paediatric retrievals of positive or suspected COVID-19 patients. *Pediatr. Res*. 10.1038/s41390-020-1050-z (2020). 10.1038/s41390-020-1050-z 32634819

[56] Nolan JP (2020). European Resuscitation Council COVID-19 guidelines executive summary. Resuscitation.

[57] Trevisanuto D (2020). Neonatal resuscitation where the mother has a suspected or confirmed novel coronavirus (SARS-CoV-2) infection: suggestion for a pragmatic action plan. Neonatology.

[58] Richards T, Scowcroft H (2020). Patient and public involvement in covid-19 policy making. BMJ.

[59] Molloy EJ, Mader S, Modi N, Gale C (2019). Parent, child and public involvement in child health research: core value not just an optional extra. Pediatr. Res..

[60] Betz, C. L. Generations X, Y, and Z. *J. Pediatr. Nurs*. **44**, A7–A8 (2019). 10.1016/j.pedn.2018.12.013 30683288

[61] Voysey M (2021). Safety and efficacy of the ChAdOx1 nCoV-19 vaccine (AZD1222) against SARS-CoV-2: an interim analysis of four randomised controlled trials in Brazil, South Africa, and the UK. Lancet.

[62] Polack FP (2020). Safety and efficacy of the BNT162b2 mRNA Covid-19 vaccine. N. Engl. J. Med..

[63] Baden LR (2021). Efficacy and safety of the mRNA-1273 SARS-CoV-2 vaccine. N. Engl. J. Med..

